# Sport-Specific Functional Tests and Related Sport Injury Risk and Occurrences in Junior Basketball and Soccer Athletes

**DOI:** 10.1155/2020/8750231

**Published:** 2020-12-11

**Authors:** Wen-Dien Chang, Chi-Cheng Lu

**Affiliations:** Department of Sport Performance, National Taiwan University of Sport, Taichung, Taiwan

## Abstract

**Objectives:**

Sport-specific functional tests were used to assess the power, speed, and agility of the lower extremity for a specific sport, but comparison of the differences and association with sport injury was rare. The aim of this study was to investigate the differences in sport-specific functional tests between junior basketball and soccer athletes and analyze the sport injury risk and occurrences.

**Methods:**

All participants were evaluated using the sprint test, vertical jump (VJ) test, agility T test, and functional movement screen (FMS). There were significant intergroup differences in the sprint test, VJ test, agility T test, and FMS. Specific functional tests were compared against FMS score, either FMS ≤ 14 or FMS > 14. The FMS subtests, namely, in-line lunge, trunk stability push-up (TSPU), and quadruped rotary stability, were also performed. In one-year follow-up, the sport injury incidence was also recorded.

**Results:**

Significant differences in sprint, agility, and FMS performance were found between the junior basketball and soccer athletes. Individual FMS scores of the in-line lunge, TSPU, and quadruped rotary stability were evaluated. No significant differences in sprint, VJ, and agility scores were found between FMS ≤ 14 and FMS > 14. FMS total score ≤ 14 was significantly associated with high sport injury occurrence.

**Conclusions:**

The scores of sprint, agility, and FMS performance were differed between basketball and soccer athletes. The scores of sprint, VJ, and agility tests did not have differences with sport injury risks and occurrences, but the FMS score was associated with sport injury occurrence.

## 1. Introduction

The age- and skill-matched controls highlighted early differentiation in junior athletes [1]. Assessing athletes' strengths and weaknesses by functional tests could help in prescribing appropriate training, conditioning, and lifestyle interventions. It is essential for optimal athlete development in the context of youth sports [2]. In professional basketball and soccer, athletes were faced with various technical demands and were also required a higher physical fitness [3]. A functional test was used to assess the combined physical fitness and technical performance. It was a sport-specific assessment focused on identifying physical fitness and match performance in a sport [4]. The sprint, jump, and agility tests were sport-specific functional tests, validating the match performances of basketball and soccer athletes. They could assess the power, speed, and agility of the lower extremity for a specific sport [3, 5]. Sprint test, vertical jump test, agility T test, and functional movement screen (FMS) were common sport-specific functional testing tools for basketball and soccer athletes [3, 5, 6]. In a previous study, the functional tests were used to assess match performances, but the usage of the tests for sport injury assessment was rare except functional movement screen [7]. Chalmers et al. have indicated that lower vertical jump, sprint, and agility were associated with an increased risk of various injuries and greater injury severity [8].

The most common ball sports among junior athletes are basketball and soccer, and these have a high rate of sport injuries in boys and girls [9]. There were a variety of sport injury factors, and intrinsic personal fitness and extrinsic environment caused sport injuries in up to 50~70% [10]. Sport development in these junior athletes should focus primarily on fitness performance and sport injury prevention. However, few studies focused on the use of sport-specific functional tests to compare the differences and association with sport injury in junior basketball and soccer athletes. More studies are also required to analyze sport-specific functional tests in various sport teams as well as the differentness between sport injury risk and occurrences in the junior athletes. The aim of this study was to investigate the differences in sport-specific functional tests for junior athletes between basketball and soccer games and analyze the sport injury risk and occurrences. We hypothesized that there are no differences in sport-specific functional tests in junior basketball and soccer athletes, but higher sport injury risk had poor functional test scores and higher sport injury occurrences.

## 2. Methods

This is a prospective observational study, and participants were enrolled from the basketball and soccer teams of two junior high schools. Junior athletes who had participated in team practice and competition for more than one year and continued to join team practice were eligible for inclusion in the study. The exclusion criteria were athletes who had sustained severe injuries; had a history of neuromuscular or cardiovascular diseases; had undergone surgery; and had visual, vestibular, or balance problems that could affect their performance. This study was approved by the institutional review board. In accordance with the study of Cosio-Lima et al. [11], the sample size was estimated to be at least 31 participants in each group. Seventy-eight participants were included in this study. The participants' data were grouped into two based on the sport terms: soccer team (Group 1, *n* = 34) and basketball team (Group 2, *n* = 44). All of them underwent four sport-specific functional tests, i.e., sprint test, VJ test, agility T test, and functional movement screen, which were used to conduct in a randomized order. At least a 20-minute break between each test was employed. A physical therapist conducted a one-on-one assessment of each participant. In one-year follow-up, the sport injury incidence was calculated via physical therapy reports of the number of athletes who suffered any sport injuries during the academic year. This study was approved by the Institutional Review Board of AT Hospital, and all participants were informed of study procedures prior to their participation.

## 3. Sport-Specific Functional Tests

### 3.1. Sprint Test

**Figure 1:**
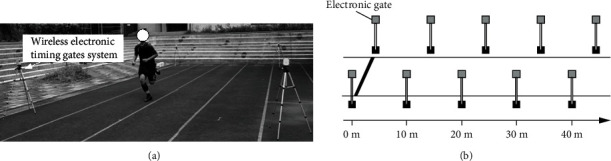
(a, b) Sprint test: 10, 20, 30, and 40 m straight-line sprints.

The wireless electronic timing gates system (Timing Gates System, You-Shang Technical Corp., Taiwan) was used for the sprint test, consisting of four distances of 10, 20, 30, and 40 m for a maximum of three straight-line sprints (Figure 1). The participants stood 0.5 m behind the starting line before they commenced each sprint, starting from a standing position. Active dynamic stretches of 3 min were performed during the warm-up. The physical therapist stood at the terminal line with a hand-held stopwatch and gave the “start” command to the participants. Tests were started with a distance of 10 m followed by a 5 min rest before the next sprint. The fastest sprint time was recorded for analysis. The sprint test has high reliability (intraclass correlation coefficient, ICC = 0.81; 90% confidence interval, CI = 0.64 ~ 0.90) and assesses the straight-line sprint performance of athletes [12].

### 3.2. Vertical Jump Test

**Figure 2:**
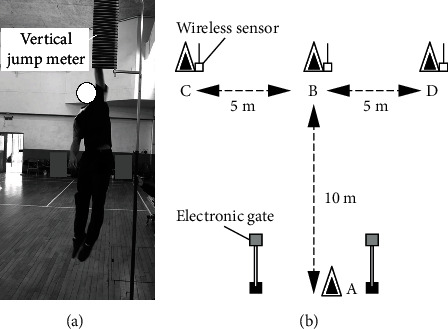
(a) Vertical jump test and (b) agility T test.

The Vertec vertical jump meter (Sports Imports Incorporated, Columbus, OH, USA) was used for the VJ test (Figure 2(a)). With their hands smeared in chalk dust, the participants stood in front of a wall and were asked to raise their hands to reach up as high as possible. The physical therapist marked the highest point reached on the wall and then asked the participants to jump vertically as high as possible to touch as high as possible on the wall. Strong verbal encouragement was provided during all tests to motivate the participants to exercise maximum effort. The difference between the two marked heights represented the VJ height. The participants performed the VJ test three times, and the highest height was selected. The VJ test is used to assess leg strength and has high reliability (ICC = 0.99; 95%CI = 098 ~ 0.99) in adolescent athletes [13].

### 3.3. Agility T Test

The wireless electronic timing system (T Test Agility Timing Systems, You-Shang Technical Corp., Taiwan) was used to measure the agility performance (Figure 2(b)). Participants were instructed to sprint forwards 10 m from cone A to cone B, touch the base of cone B, and then side shuffle 5 m to the left and touch the base of cone C. Then, they returned to the midline maintaining a forward facing position and repeated the sequence on the opposite side of the course to touch the base of cone D before running 10 m backward to the finish. The participants were asked to avoid crossing their legs during the side shuffle. Agility was determined based on the recorded time. The agility T test is used to test limb power, agility, and leg endurance and has high test-retest reliability (ICC = 0.94) [14].

### 3.4. Functional Movement Screen

**Figure 3:**
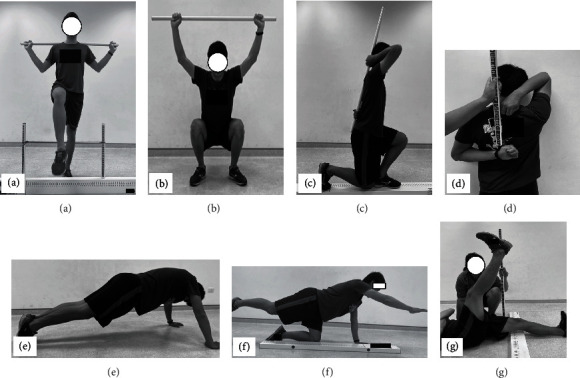
FMS test: (a) hurdle step, (b) deep squat, (c) in-line lunge, (d) shoulder mobility, (e) trunk stability push-up, (f) quadruped rotary stability, and (g) active straight leg raise.

The FMS comprises seven tests, and each test is scored on an ordinal scale of 0~3. The FMS™ (http://FunctionalMovement.com, Danville, VA, USA) tool was used to measure the functional movement performance (Figure 3). Participants should complete a deep squat, hurdle step, in-line lunge, shoulder mobility, ASLR, TSPU, and quadruped rotary stability test. If the participant performs the tested movement without any compensation, a score of 3 is given. If the movement is performed with compensation, a score of 2 is given. A score of 1 is given if the participant is unable to perform the movement, and a score of 0 is given if pain occurs during the movement. The maximum possible FMS score is 21, and a score of FMS ≤ 14 indicates a high risk of sport injury [15]. FMS is used to assess movement patterns and injury risk, and it has high interrater reliability (ICC = 0.90) [16].

### 3.5. Statistical Analysis

SPSS (version 20, IBM, NY, USA) was used for statistical analysis. Descriptive statistics for each parameter were presented as mean ± standard deviation. An independent *t*-test was used for multiple comparisons of all parameters among the two groups. High (FMS ≤ 14) or low (FMS > 14) risk of sport injury was compared in the subgroup analysis. An independent *t*-test was also used to compare the intergroup differences. Chi-square and OR were used to analyze the association with sport injury risk and occurrence. The sport injury incidence in one-year follow-up used regression analyses to assess the OR. The significance level was set at *p* < 0.05.

## 4. Results

**Figure 4:**
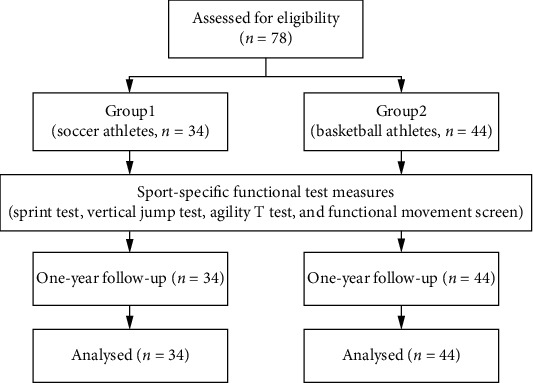
Flowchart diagram of this study.

Of the 78 adolescent athletes included for statistical analysis (Figure 4), 34 athletes were in the soccer team (age = 16.31 ± 0.13 years, height = 148.63 ± 6.37 cm, and weight = 38.73 ± 5.83 kg) and 44 athletes were in the basketball team (age = 16.28 ± 0.38 years, height = 163.63 ± 8.04 cm, and weight = 53.23 ± 11.43 kg).  Table 1 shows the timed scores of the sprint tests (30 and 40 m) and agility T test, which were significantly higher in Group 1 than in Group 2 (*p* < 0.05). The FMS total score of Group 1 was significantly lower than that of Group 2 (*p* < 0.05). Intergroup comparison revealed significant differences in the scores for in-line lunge, TSPU, and quadruped rotary stability (*p* < 0.05; Table 2). Subgroup analysis revealed no significant differences in the sprint, VJ, and agility T test scores between high (FMS total score ≤ 14) and low (FMS total score > 14) risk of sport injury (*p* < 0.05; Table 3).

During the one-year follow-up, there were 15 and 16 junior athletes in Group 1 (44.12%) and Group 2 (36.37%), respectively, who reported occurrences of sport injuries (Table 4). In Groups 1 and 2, no significant differences on sprint, VJ, and agility T test scores between noninjured and injured athletes were observed. The score of total FMS in noninjured athletes was significantly higher than that in injured athletes (Group1, *p* = 0.002; Group2, *p* = 0.02), respectively. The multivariate analysis showed no significant association among the sprint, VJ, agility T test, and FMS scores (*p* > 0.05). But the univariate analyses showed a significant association between total FMS score and the occurrence of sport injuries (Group 1, OR = 0.30, 95%CI = 0.09‐0.93, *p* = 0.03; Group 2, OR = 0.48, 95%CI = 0.23‐1.01, *p* = 0.04).

Of the 20 junior athletes with FMS total score ≤ 14 in Group 1, 13 athletes (65%) had sport injury, but 7 athletes (35%) did not have sport injury. And of the 14 junior athletes with FMS total score > 14, 4 athletes (29%) had sport injury, but 10 athletes (71%) did not have sport injury. In the soccer athletes, high sport injury risk (FMS total score ≤ 14) was significantly associated with high sport injury occurrence (OR = 0.22, 95%CI = 0.05 ~ 0.94). Of the 18 junior athletes with FMS total score ≤ 14 in Group 2, 12 athletes (89%) had sport injury, but 6 athletes (11%) did not have sport injury. And of the 26 junior athletes with FMS total score > 14, 4 athletes (15%) had sport injury, but 22 athletes (85%) did not have sport injury. In the basketball athletes, high sport injury risk (FMS total score ≤ 14) was also significantly associated with high sport injury occurrence (OR = 0.09, 95%CI = 0.02 ~ 0.39). Of all the junior athletes (*n* = 78), those with FMS total score ≤ 14 had higher sport injury occurrence (OR = 0.13, 95%CI = 0.04 ~ 0.36). The junior athletes with FMS total score ≤ 14 had higher prevalence of sport injury occurrence in Group 1 (*χ*^2^ = 4.37, *p* = 0.04) and Group 2 (*χ*^2^ = 12.09, *p* = 0.01).

## 5. Discussion

This study was designed to compare the sport-specific functional tests between junior basketball and soccer athletes and determine the sport injury risk and occurrences. Significant differences in 30 m and 40 m sprint test, agility T test, and FMS total score were found between both groups (*p* < 0.05). Comparing individual items of FMS in both groups, the items of in-line lunge, trunk stability push-up, and quadruped rotary stability had significant differences (*p* < 0.05). However, there were no significant differences in scores of the sprint test, vertical jump test, and agility T test between high and low sport injury risks in both groups. In one-year follow-up, a significant difference in total score of FMS was found between noninjured and injured athletes (*p* < 0.05), but the scores of the sprint test, vertical jump test, and agility T test did not have significant differentness (*p* > 0.05).

The current study results found that compared with adolescent soccer athletes, adolescent basketball athletes had better sport performance in physical fitness, including sprint, VJ, and agility. Basketball involves high-intensity movements, such as turning, jumping, and sprinting [17]. Soccer also requires a high level of physical fitness, especially in terms of strength, power, and speed [18]. Nonetheless, the current study found that compared with adolescent basketball athletes, adolescent soccer athletes performed poorly in sprint and agility tests.

The FMS is a popular functional testing tool and comprises seven subtests that focus on assessing competency in movement, mobility, and stability. These tests are the deep squat, hurdle step, in-line lunge, shoulder mobility, active straight leg raise (ASLR), trunk stability push-up (TSPU), and rotary stability [19]. An indicator of movement quality is the presence of asymmetry in subtests upon comparison of the movements performed on both sides of the body, which are the hurdle step, in-line lunge, shoulder mobility, ASLR, and rotary stability [19]. Moreover, the relationship between prospective injury and an FMS composite score of ≤14 has been widely reported. But Bardenett et al. thought less evidence proved that FMS composite score of ≤14 was associated with an increased sport injury risks in youth athletes of ages 8 to 21 years [20]. However, Pfeifer et al. thought that FMS is an applicable tool to identify dysfunctional movement for junior athletes [21]. FMS also could be used to predict sport injury risk for junior athletes (odds ratio, OR = 1.71) [22]. Assessing the predictive utility of FMS in junior athletes is still important, when they were still physically maturing and have potential skill development [21]. Moreover, the adolescent soccer athletes demonstrated poor functional performance on FMS assessment in the current study. The reasons for these differences in physical fitness may probably be attributed to the differential anthropometric characteristics of each sport [23]. Moreover, the differences in physical fitness could be from the differences in selection criteria of athletes for soccer and basketball [24]. Our study found the soccer athletes to have poor athletic performance. Upon analysis of the FMS subtests, the scores of the soccer athletes in three tests, i.e., the in-line lunge, TSPU, and quadruped rotary stability, were lower than those of the basketball athletes.

Movement proficiency barrier (score of individual item≦2) during FMS testing, and not the composite score, can be used to identify junior athletes at an increased risk of prospective injury. Notably, among 237 elite junior male Australian football athletes, sport injury occurrence of the athletes with movement proficiency barrier was threefold and increased in the risk of an injury to require a missed game (hazard ratio = 3.7; relative risk = 2.8) [25]. In the current study, numbers of scores of total FMS ≤ 14 were 20 (59%) and 18 (41%), and numbers of junior basketball and soccer athletes having sport injuries were 15 (44%) in soccer and 16 (36%) in basketball, respectively. We also found that average scores of individual FMS item in both groups were less than 2, and low total FMS score had higher sport injury occurrence. A previous study reported that a high percentage (65%) of elite junior Australian football athletes exhibited at least one individual item score≦2 during FMS testing [26]. The athletes who reported an injury in the previous season were 1.5 times more likely to experience pain during FMS testing, despite having no current injuries [26]. Moran et al. indicated no association has been found between dysfunctional movements of FMS composite score of ≤14 and injuries sustained by athletes in the previous season [27]. Therefore, because of the measurement bias associated with retrospective injury analysis, studies following up athletes prospectively after FMS testing are necessary [26].

The TSPU and quadruped rotary stability tests in FMS assess the static and dynamic stability of the trunk. Notably, running-based sports require trunk rotation and stability to increase sport energy transfer [28]. The agility T test and 30 and 40 m sprint tests assessed performance when running and changing directions. The soccer team was found to have poor agility and sprint performance in addition to poor functional movement in our study. Lloyd et al. analyzed the relationship between FMS and physical fitness in young soccer athletes (age = 11–16 years) [29]. They found that FMS score had a significant negative correlation with agility (*r* = −0.54, *p* < 0.05). In addition, using regression analysis, they found that the in-line lunge test is a primary predictive variable to explain agility performance (*R*^2^ = 0.46) [29]. The in-line lunge requires postural control with equal bilateral lower extremities and is both a static and dynamic task [30]. Therefore, it is a fitting indicator of agility during rapid change-of-direction movements [31]. Other studies also supported that the in-line lunge and sprint speed can be used to measure athletic performance [32, 33]. Therefore, our current study was similar with previous outcomes, resulting in poor sprint, agility, and FMS performance, especially the in-line lunge, TSPU, and quadruped rotary stability, in the junior soccer athletes.

Kiesel et al. proposed the cut-off point of FMS ≤ 14 as being more closely associated with sport injury risk [34]. The findings of Abraham et al. and Rusling et al. demonstrated that average scores of composite FMS adolescent and junior football athletes were 12.12~16.44 and were similar as our results [35, 36]. Bardenett et al. assessed FMS in 167 high school athletes (average age = 15.2 years) and found that composite FMS scores of nonsport injured and sport injured were 13.11 ± 1.69 and 13.00 ± 2.32, respectively [20]. The data of composite FMS scores were similar to that of our nonsport injured junior athletes, but differ from sport injured junior athletes. The sport characteristics of participants in the current study were basketball and soccer, and it may cause the different sport injury occurrences. A previous systematic review and meta-analysis indicated that FMS scores are relevant in sport injury occurrences [37]. Notably, an athlete with an FMS ≤ 14 was found to have 2.74 times higher risk of injury risk than those with an FMS > 14 [37]. In this study, the comparison of soccer and basketball athletes with FMS ≤ 14 and FMS > 14 revealed no significant differences in terms of sprint, VJ, and agility. The athletes with higher injury risk did not demonstrate any differences in their physical fitness compared with the athletes with lower injury risk. FMS is a cumulative score of multiple tests that can assess posture and balance deficits [38]. However, sprint, VJ, and agility performance represents gross motor skills. Classification of sport injury risk cannot observe differences in physical fitness.

The clinical implications for this study are that sport-specific functional tests could be used to compare the differences of physical fitness and sport injury in junior basketball and soccer athletes. Sprint test, VJ test, and agility T test were not applicable to identify sport injury occurrence in junior basketball and soccer athletes except FMS. Our study had some limitations. First, all the adolescent athletes were from the basketball and soccer teams of two schools, representing only a small portion of the adolescent population. Second, the severity data of sport injuries were not collected during follow-up. Therefore, future studies should adopt larger samples or involve different types of ball games along with the exploration of the occurrence of sport injury.

## 6. Conclusions

This study investigated the differences in sport-specific functional tests between junior basketball and soccer athletes and compared the differences between high and low sport injury risks. Differences in the scores of sprint, agility, and FMS performance were found between basketball and soccer athletes. Furthermore, the two kinds of athlete were found to demonstrate differences in in-line lunge, TSPU, and quadruped rotary stability of FMS, possibly because of the differential anthropometric characteristics of each specific sport. No differences in scores of sprint, VJ, and agility test were found in different sport injury risks or different sport injury occurrences. The FMS score was associated with sport injury occurrence and could be an indicator of sport injury risk.

## Figures and Tables

**Table 1:** Outcomes of sprint test, vertical jump test, agility T test, and FMS between 2 groups.

Items	Group 1 (*n* = 34)	Group 2 (*n* = 44)	*p* value
Sprint test (10 m)	2.51 ± 0.27	2.45 ± 0.18	0.47
Sprint test (20 m)	4.17 ± 0.52	3.91 ± 0.28	0.46
Sprint test (30 m)	5.74 ± 0.81	5.31 ± 0.45	0.04^∗^
Sprint test (40 m)	7.16 ± 0.74	6.70 ± 0.59	0.03^∗^
Vertical jump test	42.75 ± 6.06	44.45 ± 8.98	0.51
Agility T test	13.21 ± 1.23	12.22 ± 1.21	0.01^∗^
FMS total score	11.35 ± 2.34	13.64 ± 1.94	0.01^∗^

**Table 2:** Outcomes of 7 items in FMS between two groups.

Items	Group 1 (*n* = 34)	Group 2 (*n* = 44)	*p* value
FMS			
Deep squat	1.65 ± 0.78	1.86 ± 0.54	0.32
Hurdle step	1.52 ± 0.62	1.82 ± 0.39	0.08
In-line lunge	1.24 ± 0.83	1.73 ± 0.55	0.03^∗^
Shoulder mobility	1.87 ± 0.99	1.76 ± 0.97	0.73
Active straight leg raise	1.82 ± 0.56	1.95 ± 0.58	0.47
Trunk stability push-up	1.82 ± 1.19	2.77 ± 0.61	0.01^∗^
Quadruped rotary stability	1.41 ± 0.87	1.73 ± 0.46	0.01^∗^

**Table 3:** Comparison of sprint, vertical jump, and agility tests between FMS ≤ 14 and FMS > 14.

	Group 1		Group 2	
Items	FMS ≤ 14 (*n* = 20)	FMS > 14 (*n* = 14)	FMS ≤ 14 (*n* = 18)	FMS > 14 (*n* = 26)
Sprint test (10 m)	2.52 ± 0.22	2.40 ± 0.12	2.44 ± 0.13	2.45 ± 0.21
Sprint test (20 m)	4.23 ± 0.51	3.85 ± 0.37	3.88 ± 0.25	3.93 ± 0.30
Sprint test (30 m)	5.83 ± 0.86	5.33 ± 0.26	5.23 ± 0.43	5.36 ± 0.47
Sprint test (40 m)	7.19 ± 0.81	6.99 ± 0.21	6.58 ± 0.50	6.78 ± 0.65
Vertical jump test	42.83 ± 6.67	42.46 ± 2.15	45.25 ± 5.93	43.88 ± 10.82
Agility T test	13.24 ± 1.12	13.04 ± 1.95	12.14 ± 0.92	12.27 ± 1.41

**Table 4:** Regression analyses of sprint, vertical jump, and agility tests and FMS of sport injury.

Items	Group 1			Group 2		
	Noninjured (*n* = 19)	Injured (*n* = 15)	OR^a^	Noninjured (*n* = 28)	Injured (*n* = 16)	OR^a^
Sprint test (10 m)	2.47 ± 0.21	2.51 ± 0.31	1.77	2.44 ± 0.19	2.46 ± 0.16	2.34
Sprint test (20 m)	3.98 ± 0.33	4.30 ± 0.57	4.96	3.90 ± 0.26	3.95 ± 0.35	1.97
Sprint test (30 m)	5.58 ± 6.37	5.85 ± 0.93	1.57	5.28 ± 0.40	5.36 ± 0.59	1.44
Sprint test (40 m)	7.18 ± 0.69	7.14 ± 0.80	0.92	6.71 ± 0.59	6.68 ± 0.62	0.91
Vertical jump test	43.82 ± 1.88	42.20 ± 7.83	0.94	44.41 ± 10.21	44.53 ± 5.19	1.01
Agility T test	13.04 ± 1.26	13.33 ± 1.25	1.23	12.16 ± 1.29	12.36 ± 1.04	1.15
Total FMS	13.28 ± 1.79	10.01 ± 1.63^∗^	0.30^∗∗^	14.18 ± 1.91	12.16 ± 1.16^∗^	0.48^∗∗^

^a^Univariate analyses; ^∗^*p* < 0.05, noninjured vs. injured; ^∗∗^*p* < 0.05, significant association; OR: odds ratio.

## Data Availability

The data used to support the findings of this study are included within the article.
